# Treatment of Diphtheria

**Published:** 1887-01

**Authors:** 


Treatment of Diphtheria.—Dr. A. Brondel, in an article published in *Le Bull Gen. de Therapeutique*, describes a treatment of diphtheria which has been uniformily successful in his hands. *More than two hundred cases* have been treated without a single death, a record which seems incredible. He considers benzoate of soda a specific in this disease, but recommends sulphide of calcium in addition. The following is his formula :

| | | |
|---|---|---|
| ℞ | Sodii Benzoatis, . - - | ʒ i—ʒ i, gr. xv. |
| | Aq., - - - - | ℥ v. |

M Sig.—Tablespoonful every hour.

Calcium sulphide, gr. ⅛, is also given every hour with the potion. He also employs a spray of 10 per cent. solution of benzoate of soda given every half hour, night and day.

